# Application of Different Wavelengths of LED Lights in Antimicrobial Photodynamic Therapy for the Treatment of Periodontal Disease

**DOI:** 10.3390/antibiotics12121676

**Published:** 2023-11-28

**Authors:** Yasuo Takeuchi, Akira Aoki, Koichi Hiratsuka, Chanthoeun Chui, Akiko Ichinose, Nay Aung, Yutaro Kitanaka, Sakura Hayashi, Keita Toyoshima, Takanori Iwata, Shinich Arakawa

**Affiliations:** 1 Department of Lifetime Oral Health Care Science, Graduate School of Medical and Dental Sciences, Tokyo Medical and Dental University (TMDU), Tokyo 113-8549, Japan; shinperi@tmd.ac.jp; 2 Department of Periodontology, Graduate School of Medical and Dental Sciences, Tokyo Medical and Dental University (TMDU), Tokyo 113-8549, Japan; akiko.ichinose@hamadalab.com (A.I.); hayashi.peri@tmd.ac.jp (S.H.); toyoperi@tmd.ac.jp (K.T.); iwata.peri@tmd.ac.jp (T.I.); 3 Department of Biochemistry and Molecular Biology, Nihon University School of Dentistry at Matsudo, Chiba 271-8587, Japan; hiratsuka.koichi@nihon-u.ac.jp; 4 Flora Dental Care, Phnom Penh 120407, Cambodia; lobkob@yahoo.com; 5 Waseda Research Institute for Science and Engineering, Waseda University, Tokyo 169-8555, Japan; 6 Laser Light Dental Clinic Periodontal and Implant Center, Yangon 11241, Myanmar; nayaung.mdy@gmail.com; 7 Department of Oral Diagnosis and General Dentistry, Tokyo Medical and Dental University (TMDU), Tokyo 113-8549, Japan; kitanaka.peri@tmd.ac.jp

**Keywords:** phototherapy, antimicrobial photodynamic therapy, periodontal disease, light-emitting diode

## Abstract

Therapeutic light has been increasingly used in clinical dentistry for surgical ablation, disinfection, bio-stimulation, reduction in inflammation, and promotion of wound healing. Photodynamic therapy (PDT), a type of phototherapy, has been used to selectively destroy tumor cells. Antimicrobial PDT (a-PDT) is used to inactivate causative bacteria in infectious oral diseases, such as periodontitis. Several studies have reported that this minimally invasive technique has favorable therapeutic outcomes with a low probability of adverse effects. PDT is based on the photochemical reaction between light, a photosensitizer, and oxygen, which affects its efficacy. Low-power lasers have been predominantly used in phototherapy for periodontal treatments, while light-emitting diodes (LEDs) have received considerable attention as a novel light source in recent years. LEDs can emit broad wavelengths of light, from infrared to ultraviolet, and the lower directivity of LED light appears to be suitable for plaque control over large and complex surfaces. In addition, LED devices are small, lightweight, and less expensive than lasers. Although limited evidence exists on LED-based a-PDT for periodontitis, a-PDT using red or blue LED light could be effective in attenuating bacteria associated with periodontal diseases. LEDs have the potential to provide a new direction for light therapy in periodontics.

## 1. Introduction

**Figure 1:**
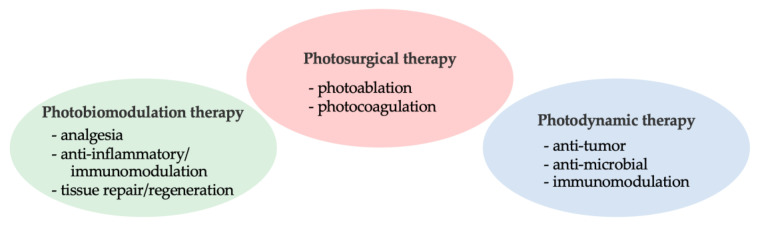
Various therapeutic effects obtained with the application of light.

Optical technology has been used in medicine for examining, diagnosing, and treating patients. Particularly in current dental treatments, it has various applications, such as surgical ablation, disinfection, bio-stimulation, suppression of inflammatory responses, and promotion of wound healing (Figure 1) [1]. Compared with conventional mechanical therapies, phototherapy, a minimally invasive treatment method, has the advantage of reducing pain, discomfort, and tissue damage. It also accelerates wound healing, thereby improving the quality of life. Antimicrobial photodynamic therapy (a-PDT) is a modern form of phototherapy, which has garnered attention as a new therapeutic method for eradicating oral bacterial infections, and its application in periodontal treatment has been investigated [2,3]. a-PDT has been predominantly performed using lasers for the treatment of periodontal infections, and its adjunctive effect when combined with non-surgical periodontal debridement has also been evaluated. Light-emitting diodes (LEDs) have been used as a new light source for a-PDT, and the number of studies on a-PDT using LEDs has increased in recent years. This review presents an overview of the light application in periodontal treatments and describes the potential application of different wavelengths of LED as a new light source in a-PDT/phototherapy for the management of periodontal diseases.

## 2. Periodontal Diseases

Periodontal disease is an inflammatory condition affecting the periodontium, which leads to the destruction of periodontal tissues and eventual tooth loss. Approximately 1.1 billion individuals worldwide are reportedly affected by severe periodontal disease [4]. Recent epidemiological, clinical, and animal model studies have suggested a possible link between periodontal disease and various systemic diseases, such as diabetes and cardiovascular disease. Preventing and controlling periodontal disease is essential for maintaining oral and systemic health [5,6,7]. Although various environmental factors and systemic conditions may be involved in the onset and progression of diseases, bacterial plaque is generally considered the primary etiological factor. Mechanical instrumentation is considered in initial periodontal therapy for eliminating plaque and calculus deposits. However, conventional mechanical therapy may not entirely eliminate these etiologic factors in areas with limited access, such as deep pockets and complicated root surfaces. Antibiotic and antiseptic chemotherapies have limited efficacy against dental plaque residing as a biofilm [8,9], and the emergence of antibiotic-resistant bacteria is concerning [10,11].

## 3. Phototherapy in Periodontics

Optical technology is widely used for treating oral diseases, especially in the field of periodontics. Lasers, a monochromatic collimated beam of light, which can be concentrated into powerful energy beams within a narrow range, have been used as light sources for phototherapy in periodontics. High-power laser devices have been used to selectively cut hard and soft tissues and further remove diseased tissues. In particular, erbium-doped yttrium aluminum garnet (Er:YAG) laser is used to remove calculus in periodontal therapy [12,13]; the laser irradiation enables minimally invasive debridement and neutralization of diseased root surfaces. Photobiomodulation (PBM) uses low-power laser irradiation to trigger photophysical and photochemical reactions in host cells, resulting in beneficial physiological changes. Several studies have demonstrated the ability of PBM to promote rapid wound healing and alleviate pain at diseased sites [14,15,16]. Photodynamic therapy (PDT), which uses light and light-activatable photosensitizers, has garnered significant interest in recent years, with an increase in the number of basic and clinical studies on PDT. 

PDT was originally developed as a treatment technique for cancers in various organs and age-related maculopathy [17,18]. PDT uses a specific wavelength of light to irradiate an area after administering a photosensitive substance, which has an affinity for the target tumor cells. The application of light excites the substance, and this process triggers the production of reactive oxygen species (ROS), leading to the destruction of the lesioned tissue. On the other hand, PDT used specifically to eradicate bacteria is known as “antimicrobial photodynamic therapy (a-PDT)”. a-PDT has been used for the neutralization of harmful substances resulting from bacterial adherence to the root surfaces [19,20]. In vitro treatment with a-PDT potently and functionally inactivates inflammatory mediators, such as tumor necrosis factor (TNF)-α and interleukin (IL)-1β, which can impair periodontal restoration; thus, photochemical reactions from a-PDT may have the potential to enhance periodontal treatment [20]. The use of a-PDT has raised concerns regarding adverse effects on host cells. However, the light intensity used for bacterial elimination or suppression is below the toxicity limit of the host cell. Moreover, the light used during a-PDT exerts photobiological effects on the surrounding tissues, thereby providing important benefits for the periodontal healing process [1]. Although the mechanism is still debated, irradiated light reduces inflammation by regulating immunocompetent cells and supports the healing of periodontal tissues [21,22,23,24,25,26,27,28,29].

a-PDT is useful for sites, which are difficult to access with mechanical instruments. Several clinical studies have examined the effectiveness of a-PDT as an adjunctive therapy after conventional mechanical debridement of periodontal disease. Diode lasers have been used as light sources for a-PDT in the majority of studies. However, the discrepancies in the application conditions of a-PDT across studies have led to some researchers reporting inconsistent results, which limit the proven effectiveness of the adjunct use of a-PDT in the treatment of periodontal disease [30,31,32]. A 2021 systematic review by Moro et al. [33] evaluated 22 studies, which used a-PDT as an adjunctive therapy to scaling and root planing (SRP) with a 3-month follow-up. The results showed that the combination of a-PDT and SRP led to a significant increase in the clinical attachment level (CAL) and a decrease in the periodontal probing depth (PD) compared with those of SRP alone. They also found that the clinical efficacy of a-PDT was high when indocyanine green or high-concentration phenothiazine chloride was used as the photosensitizer. The beneficial effects of a-PDT also include reducing periodontopathic bacteria and improving clinical parameters [2].

Laser devices have been used as light sources in phototherapy for periodontal treatments. However, the activation of the photosensitizers used in a-PDT does not necessarily require high power light of lasers, and studies employing LEDs as light sources have increased in recent years. Similar to lasers, LEDs can be applied using a fiber-type tip to effectively irradiate anatomically complex areas, such as deep periodontal pockets. The lower directivity of LED light compared with that of lasers enables the irradiation of large and complicated surfaces using a flashlight-type device. Furthermore, LED devices are small, lightweight, and less expensive than lasers, making the production of home-use LED products for daily plaque control easier.

## 4. Antimicrobial Effects of Various Wavelengths of LEDs

LED devices can output wavelengths of light in the ultraviolet (UV), visible, and infrared regions. Several major periodontopathic bacteria possess endogenous photosensitizers, which are inactivated by the wavelength of LED light irradiation alone [34,35,36]. However, more than 700 bacterial species reside in the oral cavity, and the combined use of LED lights and photosensitizers is necessary to exert an antibacterial effect against various micro-organisms in clinical practice. The antibacterial effect of a-PDT is influenced by several factors, such as the dose and type of photosensitizer, wavelength and irradiance of light, and oxygen content in the irradiation field. A higher antibacterial effect can be expected when the wavelength of the irradiation light matches the absorption wavelength of the photosensitizer. Most photosensitizers can adhere to both bacteria and periodontal tissues and exhibit toxicity to the bacteria even in the absence of light [37]. The clinical application of a-PDT in periodontal treatment requires high antibacterial effects to be accompanied by minimal damage to host cells. The number of clinical studies investigating antibacterial phototherapy using LEDs is substantially lower than that of studies using lasers; however, in vitro studies examining the antibacterial effects of various wavelengths of LED lights with and without the use of photosensitizers have been reported increasingly.

### 4.1. Search Strategy

An electronic search of PubMed/MEDLINE, Cochrane Central Register of Controlled Trials (CENTRAL), and Web of Science databases was conducted for studies in March 2023. The search was limited to literature published in English. Literature searches in these databases were performed using the following terms: (“periodontal diseases” OR “periodontitis” OR “gingivitis” OR “periodontics” OR “periodontal”) AND (“light-emitting diode” OR “light emitting diode” OR “LED”) AND (“phototherapy” OR “photodynamic” OR “photodynamic therapy” OR “antimicrobial photodynamic therapy” OR “antibacterial photodynamic therapy” OR “a-PDT” OR “PDT”). An additional manual search of the references listed in all the included articles was conducted to identify further possible articles. The literature resulting from the search was screened by the authors first on the basis of titles and abstracts, and subsequently, on the basis of full texts. Particularly in clinical studies, the full-length articles of studies were evaluated in detail for inclusion in this review, and case reports/series, studies investigating the effect of LED light irradiation alone without photosensitizer, and studies targeting the treatment of peri-implant disease were excluded. Data extracted from the clinical research papers using LED-based a-PDT listed in Table 1 included publication information, study design, sample size, photosensitizer type/concentration, irradiation parameters and regimen, investigated parameters, and follow-up period.

### 4.2. Red Light/Infrared Light

The use of red light as an excitation light in a-PDT for periodontitis has been studied extensively. Phenothiazine-based photosensitizers, such as toluidine blue and methylene blue, show absorption peaks around 600–700 nm and are frequently used in combination with red light in a-PDT for periodontal diseases. The increased proportion of Gram-negative anaerobic bacteria in the dysbiotic microbiota of the diseased sites in the periodontium and the relatively small molecular weight and structural cationic charge of these photosensitizers facilitate easy penetration into the outer membrane of both Gram-negative and Gram-positive bacteria with a high affinity.

Red/infrared diode laser was used as the light source for a-PDT with blue dye initially, and its antibacterial effect against Gram-negative periodontopathic bacteria, such as *Porphyromonas gingivalis* and *Aggregatibacter actinomycetemcomitans*, has been demonstrated in vitro. Additional studies using red/infrared LED light have been conducted to confirm their efficacy [38,39]. Commercially available laser devices specifically designed for a-PDT in dental treatment were released before LEDs, and most clinical studies have used diode lasers. The clinical efficacy of a-PDT using red or near-infrared laser light with blue dye as an adjunctive therapy to mechanical debridement has been evaluated during the non-surgical and supportive phases of periodontal therapy. Wavelengths of 660 or 810 nm (radiant exposure: 0.6 to 1414.7 J/cm^2^) were used with photosensitizers in these studies, and additional beneficial effects on clinical parameters and a reduction in the bacteriological burden were observed [2]. It has also been shown to be effective in combination with mechanical debridement during periodontal surgery [40].

**Table 1:** Clinical studies of LED-based antimicrobial photodynamic therapy in the treatment of periodontitis.

Reference	Study Participants (n) Study Design	Groups (n: Sites) Treatment Provided	Follow-Up Findings
Bassir et al., 2013 [41]	CP (16) Split-mouth	SRP + a-PDT (119): SRP (US/H) +LED (625–635 nm, 2000 mW/cm^2^, Fotosan^®^) +TB (0.1 mg/mL) Photosensitizer incubation time: not described Irradiation time: 10 s (inside) + 10 s (outside the pocket), ×6 (per tooth) SRP + LED (96): SRP (US/H) +LED (625–635 nm, 2000 mW/cm^2^, Fotosan^®^) Irradiation time: 10 s (inside) + 10 s (outside the pocket), ×6 (per tooth) SRP + PS (90): SRP (US/H) + TB (0.1 mg/mL) SRP (91): SRP (US/H)	Follow-up: 3 months. Adjunctive treatments (a-PDT, LED, or PS) were repeated 7 and 14 days later. Photoactivation using LED did not show additional effects on clinical parameters (PD, CAL, BOP, PI) compared to SRP alone.
Pulikkotil et al., 2016 [42]	CP (16) Split-mouth	SRP + a-PDT (16): SRP + LED (628 nm, Fotosan^®^) + MB Photosensitizer incubation time: 1 min Irradiation time: 10 s (inside) + 10 s (outside the pocket) SRP (16): SRP	Follow-up: 3 months. Significantly greater improvement in BOP was seen in the SRP + a-PDT group compared to that in the SRP group after 3 months of treatment. No difference in the quantity of Aa was detected between the groups.
Husejinagic et al., 2019 [43]	Periodontitis (20) Split-mouth, RCT	SRP + a-PDT (20): SRP (US/H) +LED (635 nm, 750 mW, PADPLUS) + TB (12.7 µg/mL) Photosensitizer incubation time: 1 min Irradiation time: 10 s (inside the pocket), ×6 (per tooth) SRP (20): SRP (US/H)	Follow-up: 3 months. Significant improvements in clinical parameters (PD, CAL, BOP) were shown in both groups, but the test and control groups were comparable. The recolonization of Pg and Td was reduced after adjuvant treatment, but not significantly.
Harmouche et al., 2019 [44]	Periodontitis (28) Split-mouth, RCT	SRP + a-PDT (579): SRP (US/H) +LED (625–635 nm, 2000 mW/cm^2^, Fotosan^®^) +TB (0.1 mg/mL) Photosensitizer incubation time: 1 min Irradiation time: 10–30 s (inside) + 10 s (outside the pocket) PDT applications were repeated 7 days and 3 months after SRP. SRP (609): SRP (US/H)	Follow-up: 6 months. Repeated application of a-PDT with SRP significantly improved SRP outcome (PD and BOP) compared to SRP alone. This effect was mainly observed at 6 months in initially deep sites (PD > 6 mm) with BOP.
Mongardini et al., 2014 [45]	CP (30); Residual pockets during SPT Split-mouth	SRP + a-PDT (30): SRP (H) +LED (628 nm, 2000 mW/cm^2^, Fotosan^®^) + TB (0.1 mg/mL) Photosensitizer incubation time: 1 min Irradiation time: 10 s (outside) + 10 s (inside the pocket), ×2 (per tooth) SRP (30): SRP (H)	Follow-up: 1 week. One week after the treatment, the number of sites showing a PD reduction of ≥2 mm was higher in the a-PDT group than in the SRP group. Higher reductions in relative proportions of red complex bacteria were observed in the a-PDT group compared to the SRP group.
Goh et al., 2017 [46]	Periodontitis (27); Residual pockets during SPT Split-mouth, RCT	SRP + a-PDT (36): SRP (US/H) +LED (620–640 nm, 2000–4000 mW/cm^2^, Fotosan^®^) +TB (0.1 mg/mL) Photosensitizer incubation time: not described Irradiation time: 20 s/site × 2 SRP (36): SRP (US/H)	Follow-up: 6 months. At 3 months after treatment, significantly greater improvements in PD and CAL were observed in the SRP + a-PDT group compared to the SRP group. However, the differences were no longer significant at the 6-month follow-up. Adjunctive a-PDT did not offer additional reduction in the levels of GCF cytokines, including IL-8, IL-6, and TNF-α.
Hormdee et al., 2020 [47]	Periodontitis (12) Split-mouth, RCT	SRP + a-PDT (12): SRP (US/H) +LED (420–480 nm, 1000–1200 mW/cm^2^) +CUR gel (25 µg/mg) Photosensitizer incubation time: none (irradiated immediately) Irradiation time: 2 min (inside the pocket) per tooth SRP (12): SRP (US/H)	Follow-up: 6 weeks. In the a-PDT group, significant reductions in PD and CAL were observed in the intragroup comparison from the first week up to the fourth week of follow-up. In contrast, a significant reduction in PD was observed after only a week in the SRP group. The quantities of Fn and Pi were recovered in the SRP group, while there was no significant recolonization of these bacteria on PDT sites throughout the 6-week study duration.
Ivanaga et al., 2019 [48]	CP with type 2 DM (23); Residual pockets during SPT Split-mouth, RCT	SRP + a-PDT (88): SRP (US/H) +LED (465–485 nm, 100 mW/cm^2^, InGaN) + CUR (0.1 mg/mL) Irrigation with CUR for 1 min Irradiation time: 60 s (outside the pocket) SRP + LED (80) SRP (US/H) +LED (465–485 nm, 100 mW/cm^2^, InGaN), Irrigation with 1 mL saline solution Irradiation time: 60 s (outside the pocket) SRP + PS (67): SRP (US/H) + CUR (0.1 mg/mL) SRP (97): SRP (US/H) Treatment modalities were performed in a total of 332 sites, but only 290 sites were included in the final evaluation.	Follow-up: 6 months. Significant improvements in PD and BOP were shown in all treatment groups; however, the mean values for PD, CAL, GR, BOP, and PI did not differ among the four groups at baseline, 3-, and 6-month follow-ups. Significant CAL gain was found only in the a-PDT and LED groups at 3 months in comparison to baseline data.

CP: chronic periodontitis, SRP: scaling root planing, H: hand scaling, US: ultrasonic scaling, a-PDT: antimicrobial photodynamic therapy, PS: photosensitizer, LED: light-emitting diode, MB: methylene blue, TB: toluidine blue, CUR: curcumin, PD: probing depth, CAL: clinical attachment level, GR: gingival recession, BOP: bleeding on probing, PI: plaque index, GI: gingival index, Aa: *Aggregatibacter actinomycetemcomitans*, Fn: *Fusobacterium nucleatum*, Pg: *Porphyromonas gingivalis*, Pi: *Prevotella intermedia*, Td: *Treponema denticola*, GCF: gingival crevicular fluid.

Conversely, a limited number of clinical studies have been conducted on a-PDT utilizing red LED light (Table 1). Most of these studies employed commercially available red LED devices (peak wavelength of 620–640 nm, 2000 mW/cm^2^) with photosensitizers and similar irradiation conditions [20 J/cm^2^/site × 2 from inside and outside the pocket, applying toluidine blue (0.1 mg/mL) prior to irradiation] and have demonstrated the efficacy of a-PDT as an adjunct to conventional mechanical treatment. However, several studies have reported conflicting results. For instance, Bassir et al. [41] examined the efficacy of LED-based a-PDT combined with conventional debridement in 16 patients with moderate-to-severe periodontitis and reported no additional benefits of a-PDT for the clinical outcomes compared with SRP alone. The discrepancies in the results may be due to differences in patient characteristics and a-PDT procedures (e.g., application time of photosensitizers and irradiation technique).

**Figure 2:**
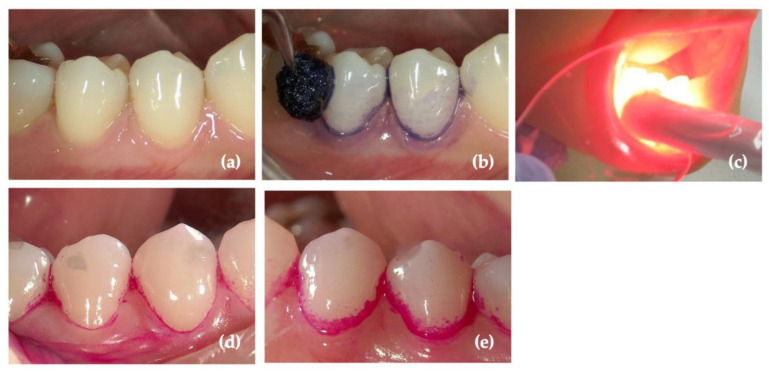
Suppression of dental plaque formation after antimicrobial photodynamic therapy (a-PDT) (red light-emitting diode (LED)/toluidine blue). Dental plaque was removed from tooth surfaces (**a**); toluidine blue O (1 mg/mL) was gently applied (**b**). After washing, red LED (660 nm, 1.1 W/cm^2^) was focused on the tooth surfaces (**c**). Suppression of plaque formation on the a-PDT group teeth (**d**) could be confirmed compared to the control teeth (**e**). Figure from Ichinose-Tsuno, A et al. Antimicrobial photodynamic therapy suppresses dental plaque formation in healthy adults: a randomized controlled clinical trial. *BMC Oral Health* **2014**, 14, 152. CC-BY 4.0 [14].

The morphology of the roots to which the bacteria are attached is complex. Periodontal pockets become deeper as periodontal disease progresses, making it increasingly difficult to align the light port with the target area even when a fiber-type tip is used. Red light is on the long-wavelength side of visible light and penetrates the gingiva to a greater extent. The penetration depth of light at the wavelength of 660 nm, which is commonly used in a-PDT, is approximately 3.0–3.5 mm [49]. Although light energy is attenuated as it passes through the gingiva [50,51], red light can act on the bacteria in the pockets, even when irradiated from the gingival surface. Transgingival irradiation facilitates easier and faster treatment without the insertion of the light into the periodontal pocket. In a study using a diode laser, Sasaki et al. [52] used a gingival model created using beef slices to determine the feasibility of transgingival a-PDT. They reported that a combination of indocyanine green-encapsulated nanoparticles (final concentration: 10 mg/mL) and infrared light from a diode laser (810 nm, 960–4800 J/cm^2^) had an antimicrobial effect even with transgingival irradiation. Schär et al. [53] evaluated the clinical effects of transgingival irradiation with red diode laser light [670 nm, 39 or 58 J/cm^2^ (single root tooth or molar)] and 1% methylene blue after SRP in periodontal pockets of patients with stage II–III periodontitis. They found that compared with the control group, the test group showed a significant reduction in bleeding on probing (BOP) and a trend toward improvement in PD and CAL after treatment. Compared with lasers, LED light is more suitable for irradiating a relatively wide area of the gingiva. The authors [54] also showed that a-PDT with red LED light (660 nm, 1.1 W/cm^2^, 20 s/site) and toluidine blue (1 mg/mL) inhibited plaque formation. Wide-area irradiation using LEDs is easy to implement and can be applied for plaque control (Figure 2).

### 4.3. Blue Light

Short-wavelength lights, such as blue light, lack the ability to penetrate deeply into tissues; thus, they cannot be used as light sources for PDT, which targets tissues deep in the human body. However, fiber-shaped tips have enabled the direct irradiation of periodontal pockets, making it possible to apply short-wavelength light for a-PDT in periodontal treatment and supportive care. Blue light can be generated by diodes or argon lasers and has been used in clinical dentistry for bleaching and curing resins. However, the equipment cost limits its widespread use. In contrast, blue LED devices are less expensive than laser devices and are now widely used to cure resin materials used for restoring dental caries. Research on the benefits of blue LED light with the aim of applying it to phototherapy is ongoing.

**Figure 3:**
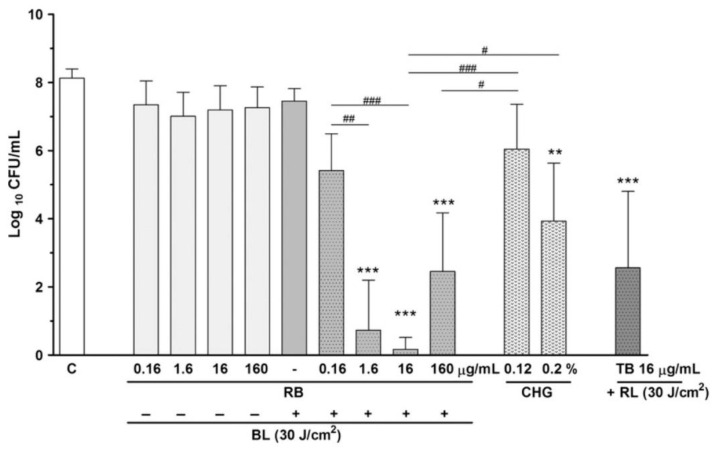
Effect of blue light-emitting diode (LED) (BL) in combination with graded rose bengal (RB). Fresh *Porphyromonas gingivalis* suspension was mixed with RB (the final concentrations were 0.16, 1.6, 16, and 160 µg/mL, respectively) and irradiated with 30 J/cm^2^ BL. Bacterial suspensions treated with chlorhexidine gluconate (CHG) or other a-PDT [a combination of toluidine blue (TB) and red LED (RL) irradiation] were employed as the positive controls. After the treatment, each suspension was plated and incubated on brucella agar plates, and the numbers of colony-forming units (CFU) were determined. a-PDT using BL and RB showed a high antibacterial effect against periodontopathic bacteria in vitro. ** *p* < 0.001, *** *p* < 0.0001 (vs. Control). ^#^ *p* < 0.05, ^##^ *p* < 0.001, ^###^ *p* < 0.0001. Figure from Chui, C et al. Antimicrobial effect of photodynamic therapy using high-power blue light-emitting diode and red-dye agent on *Porphyromonas gingivalis*. *J Periodontal Res* **2013**, 48, (6), 696–705. © Copyright (2013) John Wiley & Sons A/S. DOI: 10.1111/jre.12055 [36].

Curcumin (maximum absorption wavelength: 425 nm), riboflavin (266 nm, 373 nm, 445 nm), rose bengal (550 nm), erythrosine (530 nm), and sinoporphyrin sodium (366 nm) have been used as photosensitizers in a-PDT using third-generation blue LED light [55]. The antibacterial effect of a-PDT in combination with blue LEDs and the aforementioned photosensitizers has been demonstrated in vitro on periodontal bacteria commonly found in patients with periodontitis, such as *A. actinomycetemcomitans*, *Campylobacter rectus*, *Fusobacterium nucleatum*, *Parvimonas micra*, *P. gingivalis*, *Tannerella forsythia*, *Prevotella intermedia*, and *Streptococcus gordonii* [56,57,58,59,60,61,62,63,64,65]. In addition, the antibacterial activity of blue light irradiation alone has been demonstrated against some oral bacteria (Figure 3) [36,66,67,68,69,70,71], further MRSA, and novel coronavirus [72,73]. Black-pigmented bacteria, including *P. gingivalis* and *P. intermedia*, which are commonly associated with periodontal diseases, degrade hemoglobin to derive heme, and endogenous porphyrin is produced when the bacteria further acquire iron from heme [74]. The importance of porphyrins in the antimicrobial action of blue light has been suggested [74]; irradiated blue light reacts with endogenous porphyrin as a photosensitizer, leading to the production of ROSs, which efficiently and selectively kill bacteria [3,34,57,68,75]. This characteristic could have clinical advantages, as blue light exposure without a photosensitizer suppresses only the growth of periodontal pathogenic bacteria possessing endogenous porphyrin while simultaneously minimizing the impact on the natural microbiome. Masson-Meyers et al. [76] compared the antibacterial effects of 405 nm LED and 405 nm diode laser irradiation with the same amount of energy against *Staphylococcus aureus* and reported that both light therapies significantly suppressed bacterial growth and that the differences between the two light sources did not affect the results. Although the phases of LED light are not aligned with those of laser light, the total energy may have a greater influence on the outcome in terms of antibacterial efficacy. Blue light is more readily absorbed by the human mucosal tissue than red light [49], and excessive exposure to high-power blue light can cause tissue damage. Therefore, it is necessary to carefully consider the irradiation conditions for clinical use, although direct irradiation of the pocket can minimize the effect on host cells, which is advantageous from a safety standpoint.

Only a few clinical studies have evaluated the effects of a-PDT using blue LED (Table 1). Hormdee et al. [47] investigated the efficacy of a-PDT using blue LED and *Curcuma longa* extract as adjuncts to SRP in the treatment of patients with moderate chronic periodontitis. The application of *Curcuma longa* gel (concentration: 25 µg/mg) after SRP followed by irradiation of the pockets with blue LED (16.8 J/cm^2^) led to a significant improvement in PD and BOP at 4 weeks after treatment. The number of periodontopathic bacteria (*F. nucleatum* and *P. intermedia*) was also significantly suppressed after treatment. The probing depth decreased significantly one week after treatment in the control group; however, recurrence of the periodontal pocket with recolonization of periodontopathic bacteria was observed thereafter. Ivanaga et al. [48] investigated the clinical efficacy of a-PDT with curcumin solution (concentration: 0.1 g/L) and blue LED light (465–485 nm, 7.69 J/cm^2^, irradiated outside of the pockets) as adjunctive therapy to SRP in residual pockets after periodontal therapy in patients with periodontal disease and type 2 diabetes. A reduction in PD and BOP positivity rates was observed in the SRP-only and a-PDT combined groups after treatment. However, significant CAL gain was observed only in the a-PDT combined group at three months in comparison to baseline data. Araújo et al. [77] used a mouthwash containing a curcumin solution (concentration: 1.5 g/L) and irradiated the mouths of 13 volunteers with blue LED (450 nm, 20.1 J/cm^2^, irradiated the oral cavity by inserted light source) to determine the effectiveness of a-PDT for routine oral hygiene purposes. They examined the quantity of bacteria in the saliva before and after treatment and reported that a-PDT using curcumin mouthwash and blue LED significantly reduced the bacteria (68.3%), whereas the use of mouthwash alone did not result in an effective reduction in bacteria (9%). Ricci Donato et al. [78] instructed 50 volunteers to rinse with one of two photosensitizers (curcumin or a hematoporphyrin derivative). Light illumination (blue light at 450 nm for curcumin and red light at 630 nm for the hematoporphyrin derivative; irradiance: <100 W/cm^2^ for 6 min) was performed using a LED device. The results showed a significant reduction in the number of bacteria in the saliva after both photodynamic treatments; a reduction was observed even after 24 h of treatment in the curcumin + blue light group. Genina et al. [79] investigated the efficacy of a toothbrush equipped with a blue light-emitting function (405–420 nm, 2 mW∕cm^2^) as a daily means of suppressing the progression of periodontal disease. Sixty participants with mild-to-moderate gingivitis were randomly divided into two groups (blue LED-emitting toothbrushes and regular toothbrushes). A reduction in the accumulation of dental plaque and gingival inflammation was observed in both groups after using the toothbrushes for one month; however, the efficiency (improvement in clinical indices) of brushing was significantly higher in the LED toothbrush group than in the control group.

Only a limited number of clinical studies have been conducted on a-PDT using blue LEDs, and there is heterogeneity in study designs. Thus, it is difficult to determine its clinical efficacy at present; however, many ongoing studies have suggested a growing potential for the clinical application of blue light.

### 4.4. Green/Yellow Light

**Figure 4:**
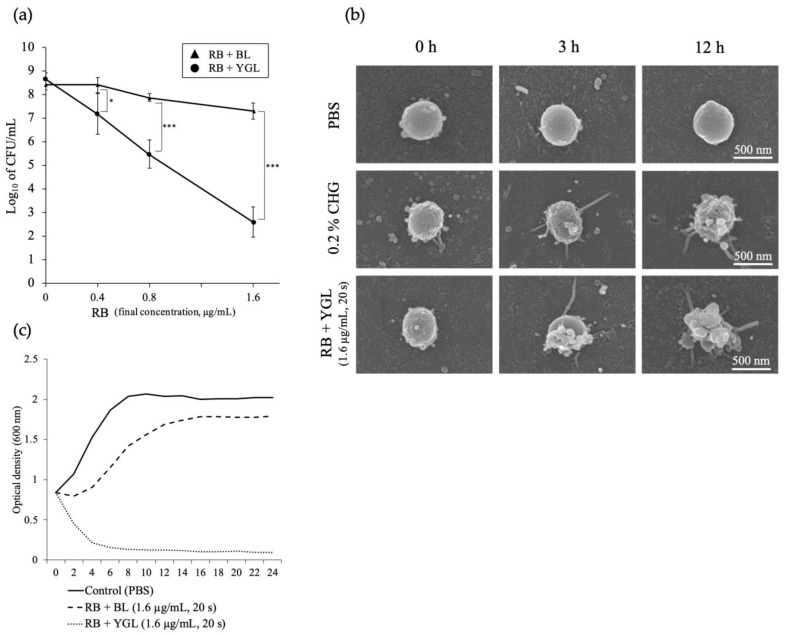
Antibacterial effects of a-PDT (rose bengal (RB) plus yellow-green light-emitting diode (LED) (YGL)) on *Porphyromonas gingivalis* (Pg). Pg suspension was mixed with RB and irradiated with YGL or BL for 20 s (8.56 J/cm^2^) in vitro. (**a**) Antimicrobial photodynamic therapy (a-PDT) treatment employing RB + YGL significantly decreased viable bacteria as compared to RB + BL. (**b**) Morphological change in Pg was noticeable after a-PDT treatment (RB and YGL). (**c**) After the treatment with RB + BL, the Pg growth was inhibited temporarily and then increased to a plateau. In contrast, a dramatic reduction in Pg growth rate in the RB + YGL group was observed up to 6 h, finally reaching the lowest plateau. * *p* < 0.05, *** *p* < 0.001. Figure from Kitanaka, Y et al. The effect of antimicrobial photodynamic therapy using yellow-green LED and rose bengal on Porphyromonas gingivalis. *Photodiagnosis Photodyn Ther* **2020**, 32, 102033. © Copyright (2020) Elsevier. DOI: 10.1016/j.pdpdt.2020.102033 [80].

Rose bengal (4,5,6,7-tetrachloro-2′,4′,5′,77-tetraiodofluorescein, RB) is present as a pigment in plaque-disclosing agents used in dentistry. a-PDT combined with RB as the photosensitizer and blue LED light (425–470 nm) exhibits high antibacterial activity against *P. gingivalis* in vitro (Figure 3) [58]. The maximum absorption wavelength of RB is approximately 550 nm. Therefore, Kitanaka et al. [80] investigated the antibacterial effects of a-PDT using a new combination of yellow-green LED light (565 nm) and RB against *P. gingivalis* in vitro. The results showed that a-PDT with yellow-green LED (8.56 J/cm^2^) and RB showed higher antibacterial activity against *P. gingivalis* than a-PDT with blue LED light (470 nm, 8.56 J/cm^2^) and RB. In addition, morphological changes suggesting leakage of bacterial contents were observed under a scanning electron microscope within a short period of time after a-PDT; no subsequent bacterial growth was observed (Figure 4). These results suggest that a-PDT with RB and yellow-green LED resulted in the physical destruction of the bacterial cell wall and high bactericidal activity. Green light has also been reported to have photobiomodulatory effects on osteoblasts and bone cells and has attracted considerable attention [81]. However, to the best of our knowledge, the effects of a-PDT using green or yellow-green wavelengths of light have not been clinically evaluated in dentistry, and this area of research is expected to progress in the future.

### 4.5. UV Light

**Figure 5:**
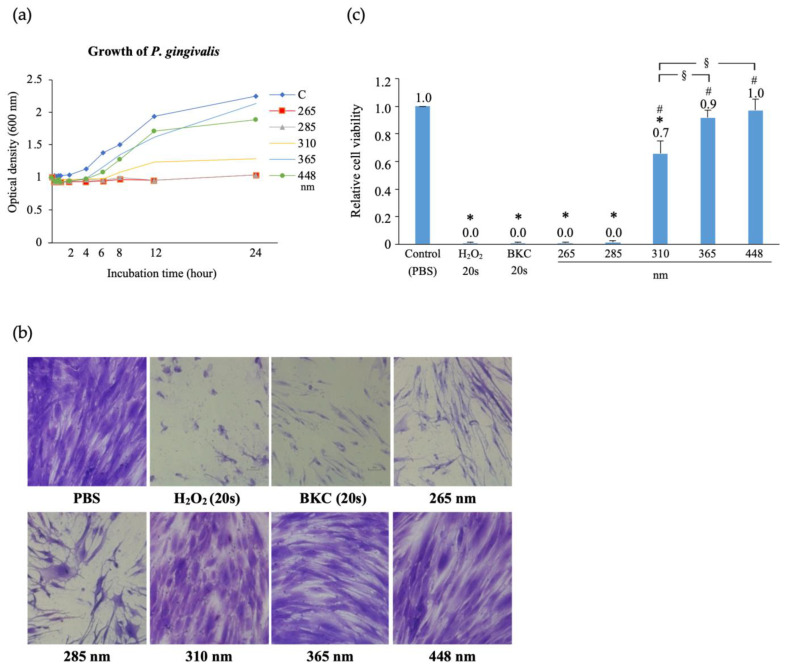
Effect of ultraviolet (UV) and blue light-emitting diode (LED) light on *Porphyromonas gingivalis* (Pg) and human gingival fibroblasts. (**a**) Growth of Pg after anaerobic irradiation (600 mJ/cm^2^) with UV and blue LED light. Pg irradiated at 265 or 285 nm showed little growth over 24 h, while a slight growth increase was observed with 310 nm irradiation. (**b**) Crystal violet staining images of human gingival fibroblasts 24 h after UV-LED irradiation. (**c**) Viability of human gingival fibroblasts 24 h after UV-LED irradiations. Oral antiseptic agents [3% H_2_O_2_ (20 s) and 0.025% benzalkonium chloride (BKC) (20 s)] and phosphate-buffered saline (PBS) were used as controls. Irradiation with UVB light (265 or 285 nm, 60 s) completely devitalized HGF-1. Irradiation at 310 nm reduced viability by 30% after 24 h, whereas irradiations at 365 and 448 nm resulted in no significant reduction compared with control group. * *p* < 0.05 (vs. Control), ^#^ *p* < 0.05 (vs. H_2_O_2_, BKC, 265, and 285 nm groups), ^§^ *p* < 0.05. Figure from Aung, N et al. The effects of ultraviolet light-emitting diodes with different wavelengths on periodontopathic bacteria in vitro. *Photobiomodul Photomed Laser Surg* **2019**, 37, (5), 288–297. © Copyright (2019) Mary Ann Liebert, Inc. DOI: 10.1089/photob.2018.4514 [86].

The peak of light absorption for DNA is at 260 nm. UVC (200–280 nm) and UVB (280–320 nm) can induce the formation of pyrimidine-pyrimidone (6-4) photoproducts or cyclobutane-type pyrimidine dimers, which damage DNA [82,83,84]. The bactericidal effect of UV light on oral bacteria is thought to be mediated via a similar mechanism. In vitro studies have shown that UV-LED light has a bactericidal effect against oral bacteria, such as *F. nucleatum*, *P. gingivalis*, *S. mutans*, and *S. sanguinis* [85,86]. The antibacterial action of UV light alone (without the use of a photosensitizer) was evaluated in these studies (Figure 5). UVC-LED showed a strong bactericidal effect; however, high cytotoxicity was demonstrated in a cell viability test using gingival fibroblasts [86]. UVB-LED exhibited a weaker bactericidal effect on oral bacteria than UVC-LED; however, it showed lower cytotoxicity to gingival epithelial cells. UVB-LED may induce the production of ROS from oral epithelial cells and enhance bactericidal activity against specific periodontopathic bacteria, such as *P. gingivalis* [85]. These results suggest that UVB-LED can also be used to control infections in the oral cavity.

Narrow-band (NB)-UVB light, the specific wavelength around 310 nm with a narrow peak, has already been used to treat skin diseases—including psoriasis, vitiligo vulgaris, and atopic eczema—in dermatology. NB-UVB has fewer side effects on the host than broad-band-UVB light [87,88,89] and has been reported to upregulate regulatory T cells [90]. The immunosuppressive effects of NB-UVB may also be preferable for the treatment of periodontitis [91,92,93,94]. However, at present, few studies have investigated the application of UVB-LED light in a-PDT or phototherapy for periodontal treatments, and the efficacy and safety should be carefully investigated through in vitro and clinical trials before applying it in clinical practice. UVB-induced immunosuppression may facilitate oral microbial infection. Over-irradiation with UVB light can cause damage to oral tissues and increase the risk of oral cancer [95,96].

## 5. Future Perspectives and Conclusions

Mechanical debridement at the site of infection remains the gold standard of treatment for periodontal diseases. However, phototherapy may be a useful adjunct to conventional therapy in anatomically complex areas and areas with limited access. a-PDT is a minimally invasive and virtually painless treatment, which does not produce antibiotic-resistant bacteria.

Although the irradiance of LEDs is generally low compared with that of lasers, a certain low threshold of light energy may be sufficient to induce bactericidal effects using a-PDT. LEDs generally have a low heat output; however, when used at high irradiance during treatment, the excessive heat generated by LEDs causes discomfort to patients. Moreover, there is a possibility of phototoxicity to neighboring tissues, which are part of the irradiation field. Therefore, further in vitro, in vivo, and clinical studies are required to determine the optimal irradiance, irradiation time, and other aspects to ensure the safety and effectiveness of LED phototherapy. To promote the clinical application of LED-based a-PDT, it is necessary to perform in vitro experiments under standardized irradiation conditions and establish recommended a-PDT protocols, which are presumed to be highly effective based on basic research data.

The wavelength of the irradiated light should be matched with the wavelength, which can be absorbed by the photosensitizer, particularly when performing a-PDT. LEDs produce light with various peak wavelengths within a narrow bandwidth. Thus, LEDs can provide light at an optimal wavelength for any photosensitizer (including endogenous photosensitizers in the pathogenic bacterial cells) used in phototherapy. However, simply increasing the dose of light or the concentration of a photosensitizer may not improve the effect of a-PDT [97,98]. Photochemical reactions and the characteristics of photosensitizers (i.e., the toxicity of the photosensitizer itself) also affect the results of a-PDT. A higher concentration of the dye can cause dimerization or multimerization of the photosensitizer and shift the absorption wavelength peak of the dye. The mismatch between the wavelengths of the light and the photosensitizer limits the production of ROS, resulting in attenuation of the antibacterial effect of a-PDT. At present, no consensus exists on the optimal setting of photosensitizers in a-PDT in periodontal practice (i.e., concentration, incubation time, etc.). Indeed, various concentrations of photosensitizers have been used for periodontal treatment of a-PDT (i.e., methylene blue: 0.005–1%; toluidine blue: 0.1–0.5%; and indocyanine green: 0.025–0.5%) [2]. Meanwhile, in most of the previous studies, the incubation time of the photosensitizers was set to 1 min, and the antibacterial efficacy of a-PDT was evaluated. A longer incubation time would increase the amounts of photosensitizers on the bacterial cells, resulting in strong antibacterial effects. However, the cytotoxicity of photosensitizers themselves has also been demonstrated in in vitro studies, and the incubation period would preferably be the minimum time required for the photosensitizers to be deposited on the target bacteria. Further in vitro and clinical studies should be conducted to determine this.

Inflammatory periodontal destruction is caused by dysbiotic polymicrobial communities, and at least 30–100 species are generally identified from a single periodontal pocket [99]. Recent research on the periodontal microbiota has clarified how the interactions between specialized community members determine an emergent overall function, which promotes or destabilizes periodontal tissue homeostasis. For example, although they are present in low numbers, keystone pathogens contribute to the emergence of dysbiotic microbiota by subverting the host immune response [100]. The appropriate wavelength of light, characteristics of the photosensitizer, and dose of light required to kill the bacteria vary with the species, and it may be necessary to determine the species or specific group of bacteria, which will be the therapeutic target of a-PDT.

Increasing the bactericidal effect is not the only way to enhance the therapeutic efficacy of a-PDT. Some wavelengths of light can activate host cells; this is known as PBM therapy [101,102,103]. The combination of multiple narrow wavelengths and intensities of LED light may be used as a new light source with high antibacterial properties and low toxicity to host cells, thereby increasing the effectiveness of periodontal treatment [104]. In addition, concurrent mechanical debridement of plaque biofilms and dental calculus will increase the effectiveness of a-PDT in treating periodontal disease. Bacteria surrounded by a biofilm matrix are less active and can withstand starvation and other harsh conditions. It is difficult for photosensitizers to penetrate biofilms, and in vitro studies have reported that biofilm-formed bacteria are more resistant to a-PDT than planktonic bacteria [35,105,106,107]. Although clinical validation is required, repeated application of a-PDT may also be effective [108,109].

The number of clinical trials using LED-based a-PDT for periodontitis is limited. Moreover, there is also heterogeneity in the study designs, which makes it difficult to compare their results. Nevertheless, previous research suggests that a-PDT using red or blue LED light appears effective in attenuating bacteria associated with periodontal diseases. Further randomized controlled trials with a standardized application protocol should be conducted to promote the clinical use of LED-based a-PDT. Progress in this research will increase the precision and reliability of these therapies in periodontal practice. LED devices have attractive features, which lasers do not possess. Thus, LEDs have the potential to provide a new direction for light therapy in periodontics.

## Data Availability

No new data were created or analyzed in this study. Data sharing is not applicable to this article.
